# Intact sensorimotor rhythm abilities but altered audiovisual integration in cochlear implant users

**DOI:** 10.1371/journal.pone.0320815

**Published:** 2025-04-02

**Authors:** Olivier Valentin, Nicholas Elgin Vernam Foster, Bastien Intartaglia, Marie-Anne Prud’homme, Marc Schönwiesner, Sylvie Nozaradan, Alexandre Lehmann

**Affiliations:** 1 School of Communication Sciences and Disorders, Faculty of Health, Dalhousie University, Halifax, Nova Scotia, Canada; 2 Laboratory for Brain, Music and Sound Research & Centre for Research on Brain, Language or Music, Montreal, Quebec, Canada; 3 Centre for Interdisciplinary Research in Music Media and Technology, Montreal, Quebec, Canada; 4 Department of Otolaryngology–Head and Neck Surgery, Faculty of Medicine, McGill University, Montreal, Quebec, Canada; 5 Research Institute of the McGill University Health Centre, Montreal, Quebec, Canada; 6 International Max Planck Research School on Neuroscience of Communication, Leipzig University, Leipzig, Germany; 7 Institute of Neuroscience (IONS), Université catholique de Louvain, Louvain-la-Neuve, Belgique; University of Hamburg, GERMANY

## Abstract

Perception of rhythm significantly impacts various aspects of daily life, including engaging with music, discerning speech prosody nuances, and coordinating physical activities like walking and sports. Numerous studies in cognitive sciences have highlighted that human rhythmic synchronization is more precise when responding to auditory rhythmic stimuli than to visual ones when the timing cues are identical. However, deaf individuals were shown to display a heightened proficiency in synchronizing their movements with visual timing cues, outperforming hearing controls (HC). Furthermore, it was demonstrated that cochlear implant (CI) users can synchronize their movements with the rhythm of unpitched drum tones. These findings raise an important question: do CI users possess a visual synchronization advantage from their pre-implant deafness, while maintaining auditory synchronization skills comparable to those of HC? Alternatively, does the neural reorganization post-implantation negate the visual synchronization advantage acquired before the implant? This study aims to answer these questions by using a sensorimotor synchronization task to probe multisensory processing abilities in CI users. Specifically, we assessed unimodal and multimodal auditory and visual abilities in CI users compared to HC using a finger tapping synchrony task with four isochronous stimulus conditions: an auditory metronome, a visual metronome, a synchronous presentation of both the auditory and visual metronomes at the same tempo, and an asynchronous presentation of the auditory and visual stimuli at differing tempos. Synchronization to auditory stimuli surpassed synchronization to visual stimuli in both groups. CI users and HC demonstrated similar unisensory synchronization consistency within the visual and auditory conditions. While HC enhanced their consistency in the audio-visual synchronous condition compared to the unisensory visual condition, CI users did not display the same improvement. Furthermore, the interference from incongruent auditory information in the asynchronous condition was comparable in HC and CI users. This study highlights that, although pitch processing is known to be impaired in CI users, our findings suggest that rhythm processing remains relatively spared. As anticipated, CI users demonstrate similar auditory rhythmic synchronization skills to those of HC, in line with existing research. Moreover, we find that, unlike deaf individuals, CI users do not exhibit an advantage in visual rhythmic synchronization, which may be due to the relatively few CI users in the study who had early prolonged pre-implantation deafness. The observed shift in audio-visual integration among CI users suggests that post-deafness or post-implantation reorganization of their auditory cortex may impede the effective integration of temporal auditory stimulation from the implant and visual information.

## Introduction

In music, rhythm provides a structural foundation that guides both musicians and listeners through the progression of a composition [1]. Rhythm is also a powerful tool for evoking emotions and engaging the audience, as rhythmic patterns can convey a wide range of emotions [2]. Rhythm in music also infuses in people the desire to synchronize their movement with the beat, adding a physical dimension to their music experience [3].

In speech, rhythm patterns facilitate in the communication and comprehension of language [4]. Rhythm patterns play a crucial role in phonological awareness, helping individuals to distinguish speech sounds and syllables, which is fundamental for language acquisition and reading development, especially in early childhood [5]. Rhythm variations in speech allow individuals to convey nuanced meaning and emotions effectively [6].

Beyond music and speech, rhythm is also integral to many daily physical activities. Rhythm provides a sense of timing and coordination that is essential for reaction time and decision-making in fast-paced sports and dynamic situations [7]. Additionally, rhythm plays a key role in motor skill development, especially during the early acquisition of walking, thanks to its ability to enhance balance and stability [8].

Rhythmic synchronization has been extensively studied in the past [7]. Interestingly, a consistent finding emerges from these studies: humans exhibit greater precision in synchronizing their movements with auditory rhythmic stimuli compared to with visual ones. This advantage is attributed to the superior temporal processing capabilities inherent to the auditory system, along with the distinct coupling that exists between auditory and motor systems [9]. However, this auditory advantage is not absolute and can be influenced by experience and stimulus characteristics [10–11]. Moreover, a study conducted by Iversen et al. in 2015 revealed that deaf individuals outperformed hearing controls (HC) in synchronizing their movements with visual timing cues [12]. Concurrently, another study performed by Phillips-Silver et al. in 2015 showed that cochlear implant (CI) users were able to move in time to the beat of music, although not as well as HC [13].

The current study aims to investigate whether CI users exhibit a visual synchronization advantage akin to that observed in deaf individuals, while also maintaining auditory synchronization skills comparable to those of HC. Specifically, we assessed unimodal and multimodal auditory and visual abilities in CI users compared to HC using a standard paradigm of one-to-one sensorimotor synchronization to periodic inputs. To assess the simultaneous integration of sensory inputs, temporally congruent audio-visual stimuli were used to determine how CI users and HC process information presented concurrently, while incongruent audio-visual stimuli were used to explore potential distinctions between CI users and HC in resolving interference arising from conflicting sensory inputs [14]. We hypothesized that, due to the hearing restoration from their implants, CI users would demonstrate synchronization consistency in the unisensory auditory condition comparable to that of HC. We postulated that CI users would not exhibit a synchronization advantage in the unisensory visual condition, either due to insufficient pre-implantation deafness duration, or as a result of post-implantation neural plasticity reversal.

## Methods

### Participants

The data presented in this study were collected between July 21^st^, 2015, and December 9, 2017. Twenty adult cochlear implant (CI) users with a mean age of 43.2 years (SD 15.0; 15 females) and seventeen paired hearing controls (HC) who were age- and gender-matched (mean age of 41.1 years; SD 15.5 years; 12 females) were recruited for this study. Three CI users did not have a pairwise HC match; there were no group differences on age or gender balance. CI users were recruited through the Raymond-Dewar Institute (Montreal, QC, Canada) and the MAB-MacKay Rehabilitation Center (Montreal, QC, Canada), two centers offering rehabilitation programs for the hard-of-hearing individuals. Table 1 details the clinical characteristics of the CI participants. All participants provided written informed consent in the study and were compensated for their participation. The study was approved by the Research Ethics Board of the Centre for Interdisciplinary Research in Rehabilitation of Greater Montreal (CRIR-985–0714).

**Table 1:** Clinical characteristics of CI group participants.

ID	Age	Age at onset of deafness	Pre-CI deafness duration	Age at implantation	CI use duration
1	22	pre-linguistic	8	8	14
2	22	2	2	4	18
3	43	6	30	36	7
4	51	37	2	39	12
5	19	7	3	10	9
6	29	2	20	22	7
7	63	40	20	60	3
9	35	24	2	26	9
10	49	42	1	43	6
11	56	49	4	53	3
12	33	pre-linguistic	16	16	17
15	65	31	32	63	2
16	47	33	6	39	8
18	41	21	3	24	17
19	57	20	33	53	4
20	43	19	19	38	5
21	20	1	18	19	1
22	58	48	7	55	3
23	58	40	15	55	3
25	52	25	26	51	1

### Stimuli

#### Auditory stimulation.

The auditory stimulus consisted of a metronome sequence containing a repeated 6 ms broadband percussive sound (200 Hz - 10 kHz) with a total sequence duration of 39.5 sec. The rate of the metronome was 2.4 Hz. The stimulus was generated using Matlab R2007a (MathWorks, Natick, MA, USA) and presented with two 8040A bi-amplified loudspeakers (Genelec, Natick, MA, USA) located at 1.5m on each side of the participant at a global sound pressure level (SPL) of 70 dB.

#### Visual stimulation.

The visual stimulus was produced by a square matrix (3.7 cm x 3.7 cm) of blue light-emitting diodes located in front of the subjects at a distance of one meter. The LED square produced flashes at a frequency of 2.4Hz (15 ms ON time) for the visual-only and synchronous audio-visual conditions. In the asynchronous audio-visual condition, the frequency was 2.6Hz. A RX6 signal processing system (Tucker Davis Technologies, Alachua, FL, USA) was used for sub-millisecond precision of stimulus presentation.

### Task procedure

**Fig 1:**
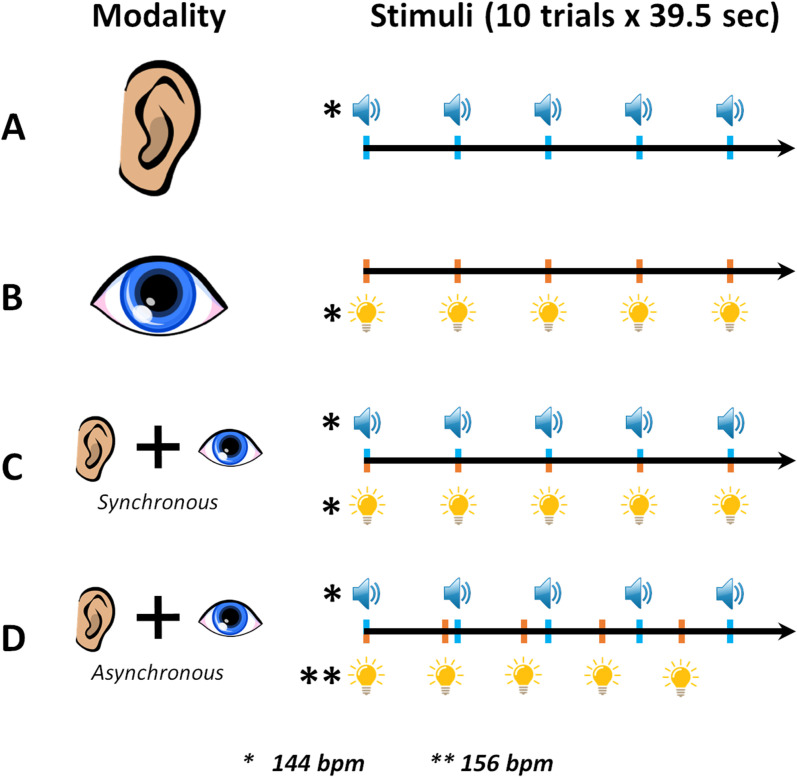
Experimental condition matrix for auditory and visual synchronization tasks.

The experiment was divided into four blocks, each consisting of 20 trials. The first two blocks contained detection tasks which will not be discussed in this paper. The third block included 10 trials from each of the two single modality synchronization conditions, and the fourth included 10 trials from each of the two multi-modal synchronization conditions. There were four different conditions: auditory-only (condition A), visual-only (condition B), synchronous audio-visual (condition C) and asynchronous audio-visual (condition D). Trials within each block were either A and B trials in a randomized order, or C and D trials in a randomized order. See Fig 1 for a detailed condition matrix. The presentation order of the trials was randomized within each block. Participants were instructed to tap their finger once every two beats while synchronizing with the sequence. They were specifically instructed to initiate their taps on the 3rd beat, providing time for participants to begin perceptually tracking the beat before they started tapping. The experiment was self-paced, and participants had to press the ‘enter’ key on a keyboard to launch the next trial. Throughout the task, regardless of stimulus modality, participants were instructed to maintain their gaze on the center of the LED-square designed for presentation of the visual stimuli. Participants were also instructed to lift their index finger from the sensor between each tap, and to use the pad of their finger rather than the nail, when tapping. There was a mandatory break after the 10th trial. Participants’ tap responses were recorded using a force-sensitive resistor (FSR) interfaced with the RX6 signal processing system, while sitting comfortably inside a double-walled audiometric booth.

### Data preprocessing

Tap response latencies were determined from the recorded FSR signal using a threshold-crossing algorithm (*RLW_events_level_trigger* function from the LetsWave 6 Matlab toolbox [15]). A technical malfunction in the FSR signal recording code resulted in a single 1.46s period of signal dropout at a variable location in most trials. To guard against incorrect tap detection time at the end of this period, any detected tap that was within a particular time window after the dropout was discarded; this window was 75% of the instructed inter-tap interval, i.e., 750ms x (2/2.4) =  625 ms. Additionally, each trial was manually inspected to remove any double taps or noise that had been flagged as a tap. All these countermeasures were effective in mitigating the impact of the signal dropout and ensured reliability in the results.

Although participants were instructed to begin tapping on the third stimulus and then continue tapping every other stimulus (i.e., on the odd stimuli), in practice the participants often ended up tapping on the even stimuli, and on occasional trials tapped at a rate of 1x or 3x the stimulus interval. For this reason, on each trial the overall tapping interval was detected based on the nearest stimulus interval to the trial’s median inter-tap interval, and trials in which participants tapped every stimulus or every third stimulus were excluded from analysis (number of trials excluded for HC: auditory-only 6, visual-only 18, synchronous audio-visual 17, asynchronous audio-visual 4; for CI users: auditory-only 25, visual-only 13, synchronous audio-visual 33, asynchronous audio-visual 17). The overall tapping phase (tapping on the odd or even stimuli) was detected by convolving the stimulus times (odd or even) and response times with a gaussian function, calculating the resulting correlation between stimulus and response convolved series, and selecting the phase (even or odd) having greater correlation. To allow participants to reach a stable state of synchronization, the first 5 response taps were discarded. Additionally, pauses during tapping were identified by calculating inter-tap intervals and detecting outliers (third quartile plus 3x the inter-quartile range); the tap at the end of any such outlier intervals was discarded.

### Data scoring

Synchronization consistency was assessed from tap latencies using circular statistics [16], providing a measure of synchronization consistency/precision (vector length) and synchronization accuracy (vector direction) for each trial, using a comparable methodology as the one described in previous work from Iversen *et al.* [12] and Nozaradan *et al.* [17]. The choice to use circular statistics allowed us to focus on synchronization consistency for analysis, in contrast to asynchrony-based measures that conflate consistency and accuracy (i.e., phase preference). Tap latencies were converted to vector angles on a unit circle. The full diameter of the unit circle corresponds to one “beat”, i.e., twice the inter-stimulus interval with respect to the even or odd stimuli as detected in the previous step. Tapping consistency is measured by calculating the mean resultant vector length. The vector length varies between 0 and 1, with lower scores (near 0) representing lower precision and high scores (near 1) higher precision. Prior to analysis, vector length values underwent a logit transformation to reduce data skewness, which is typical of synchronization data [18,19].

### Analyses

Statistical analysis was performed using R Statistical Software (3.6.1.; R Core Team 2019, RRID:SCR_001905) [20]. A mixed-effects model was used to test the effects of group and task condition on tapping consistency scores (logit vector length values), using lmerTest (Kuznetsova et al., 2017; RRID:SCR_015656) [21] with lme4 (Bates et al., 2015; RRID:SCR_015654) [22]. Group and condition were parameterized using sum-to-zero effects coding, and model terms were entered for their main effects and interaction. A random effect of participant was added to account for the hierarchical structure of the data, and a random slope for condition was added to account for unequal variance in scores across conditions. Omnibus tests of the main effects and their interaction were performed on this model via the car package [23], yielding F and p values calculated from Wald tests using Kenward-Roger approximated degrees of freedom [24] and type 3 sum of squares. Following a significant interaction effect, the emmeans R package [25] was used to test post-hoc contrasts for the following comparisons of interest: unisensory visual synchronization between groups (V_CI_ – V_HC_); unisensory auditory synchronization between groups (A_CI_ – A_HC_); difference between unisensory visual and auditory synchronization within groups (i.e., potential auditory advantage; A_CI_ - V_CI_ and A_HC_ - V_HC_); difference in unisensory auditory advantage (auditory-visual difference) between groups ((A_CI_ – V_CI_) - (A_HC_ – V_HC_)); multisensory congruence effect of synchronous auditory stimulus compared to unisensory visual stimulus within groups (AVsync_CI_ – V_CI_ and AVsync_HC_ - V_HC_); difference between groups in multisensory effect of synchronous auditory stimulus compared to unisensory visual stimulus ((AVsync_CI_ – V_CI_) - (AVsync_HC_ - V_HC_)); multisensory effect of interfering asynchronous auditory stimulus compared to unisensory visual stimulus within groups (AVasync_CI_ – V_CI_ and AVasync_HC_ - V_HC_); difference between groups in multisensory effect of interfering asynchronous auditory stimulus compared to unisensory visual stimulus ((AVasync_CI_ – V_CI_) - (AVasync_HC_ - V_HC_)). A Bonferroni correction for 11 multiple comparisons was applied to the results of these contrasts.

To explore the contribution of the onset and duration of pre-implantation deafness to task consistency among CI users, linear regressions were performed with the dependent variables of unisensory visual consistency (V_CI_), unisensory auditory consistency (A_CI_), and the multisensory congruence effect of synchronous auditory stimulus compared to unisensory visual stimulus (AVsync_CI_ – V_CI_). For each of these dependent variables, separate regressions were tested for the following predictors: age of pre-implantation deafness in years (for participants who were recorded as “pre-linguistic”, the value of 2 years was entered in the regression), and duration of deafness in years.

## Results

**Fig 2:**
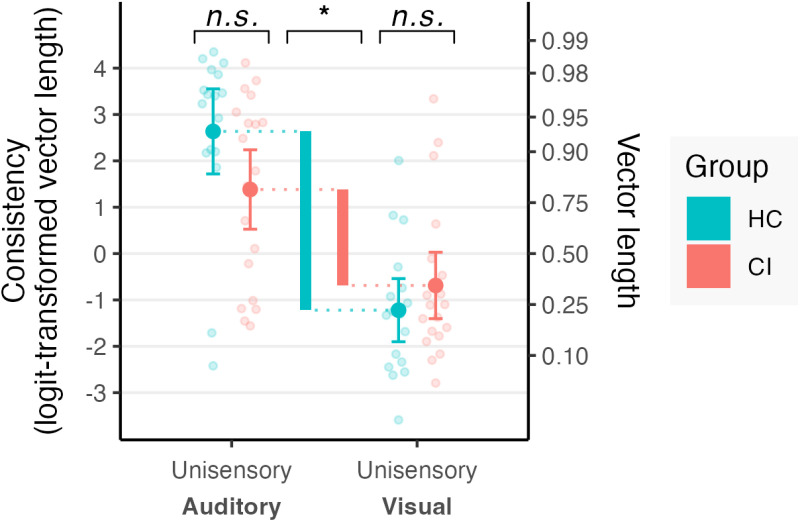
Synchronization consistency in the unisensory auditory and visual conditions. Thick bars indicate the consistency difference between auditory and visual conditions for each group. Analyses were performed on logit-transformed circular vector length; untransformed vector length values are indicated on the right axis for comparison. Higher values represent greater synchronization consistency.

A summary of performance by condition and group is provided in Table 2. Synchronization consistency varied by group and task condition (group x task interaction, F(4, 32.923) =  4.2137, p = .013). The results are presented on the basis of post-hoc contrasts examining specific comparisons of interest. Fig 2 shows the unisensory synchronization consistency results obtained with auditory and visual stimuli.

**Table 2:** Synchronization consistency scores by condition and group.

	Hearing controls			CI users		
Condition	N	Mean	SD	N	Mean	SD
Auditory-only	17	2.64	1.93	20	1.38	1.95
Visual-only	17	-1.22	1.43	20	-0.69	1.63
Synchronous audio-visual	17	1.71	1.78	20	0.20	1.85
Asynchronous audio-visual	17	-2.16	0.39	20	-1.72	0.40

Both CI users and HC exhibited superior synchronization to auditory stimuli compared to visual stimuli (CI: t(34.83) =  4.67, p < .001; HC: t(35.18) =  7.99, p < .001). Consistency on the visual condition did not differ significantly between the two groups (t(35.01) =  1.04, p ≅  1); nor did consistency differ between groups in the auditory condition (t(35.01) =  -1.97, p = .630). A trend towards greater advantage for auditory over visual synchronization was observed in HC compared to CI, although this trend did not reach statistical significance following Bonferroni correction (t(35.02) =  -2.72, p = .110).

**Fig 3:**
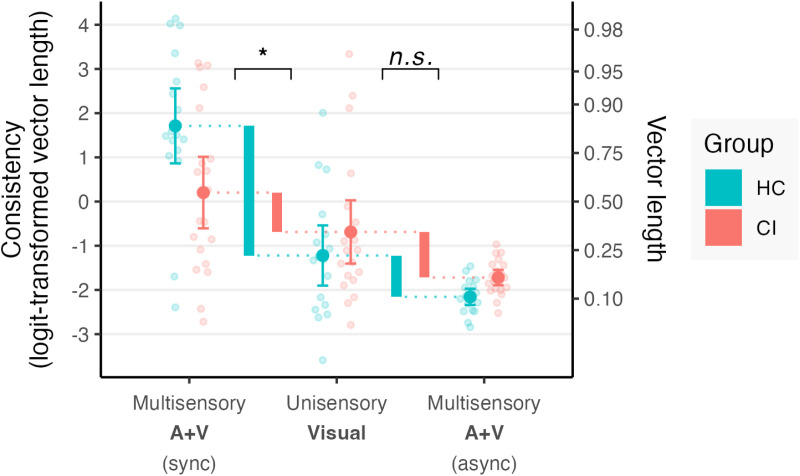
Synchronization consistency in the multisensory auditory-visual conditions as compared to the unisensory visual condition. The left pair of thick bars indicates the increase in consistency when synchronous (congruent) auditory stimulation was added, compared to visual-only, in each group. The right pair of thick bars indicates the decrease in consistency when asynchronous (incongruent) auditory stimulation was added. Analyses were performed on logit-transformed circular vector length; untransformed vector length values are indicated on the right axis for comparison. Higher values represent greater synchronization consistency. Bars represent 95% confidence intervals of the mean.

Fig 3 presents the multisensory consistency results obtained in synchronous and asynchronous conditions. When the auditory timing was congruent with visual timing (i.e., synchronous condition), HC demonstrated improved consistency compared to unisensory visual timing (t(35.14) =  6.56, p < .001), while CI users did not exhibit a better consistency in this condition (t(34.72) =  2.14, p = .438); in a direct comparison, this multisensory congruence effect was greater in HC than CI users (t(34.95) =  -3.39, p = .019). In the asynchronous condition, the impact of incongruent auditory information was similar for both groups (t(34.95) =  0.28, p ≅  1).

**Fig 4:**
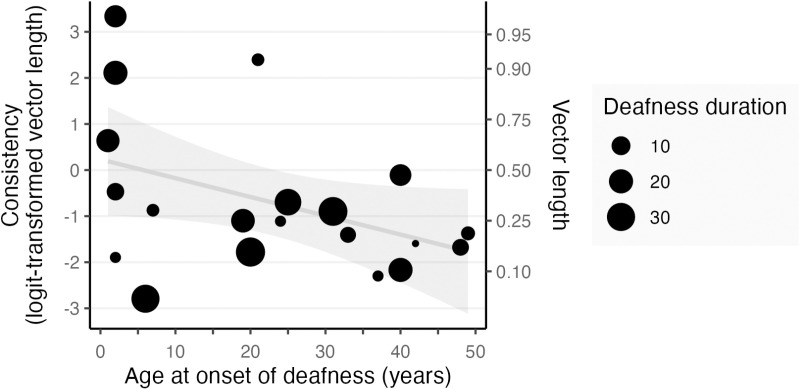
Synchronization consistency in the unisensory visual condition in CI users compared to the age and duration of pre-implantation deafness (both in years). Consistency values below ~  -0.8 can be considered chance performance (i.e., non-significant circular Rayleigh test). Regression of consistency vs onset age: b =  -0.04, t(18) =  -1.97, p = .06. Bars represent 95% confidence intervals of the mean.

Regression analyses within the CI user group found a marginal, non-significant relation between unisensory visual consistency and the onset age of deafness (b =  -0.04, t(18) =  -1.97, p = .06). As shown in Fig 4, it may be noted that although CI users whose deafness began after the age of 25 generally exhibited low visual synchronization consistency regardless of duration of deafness, those who had a combination of both very early and prolonged deafness are among those with the highest consistency on the unisensory visual condition. No relation was found between consistency on the unisensory visual condition and years of pre-implantation deafness (t(18) =  -0.02, p = .986), between consistency on the unisensory auditory condition and deafness age of onset (t(18) =  0.30, p = .769) or duration (t(18) =  -1.27, p = .219), nor between the multisensory congruence effect and deafness age of onset (t(18) =  1.16, p = .262) or duration (t(18) =  -0.74, p = .467).

## Discussion

This study investigated the integration of auditory and visual sensory modalities in CI users and HC during rhythmic synchronization tasks. The existing literature has emphasized the advantage of auditory stimuli over visual stimuli in rhythmic synchronization [26–29]. Our results find that this advantage holds in CI users for unisensory synchronization, but also reveal a nuanced pattern of cross-modal congruence and incongruence effects on synchronization consistency between CI users and HC.

As anticipated, both CI users and HC demonstrated superior consistency in auditory synchronization when compared to visual synchronization. This result is in line with previous research reporting weaker consistency at synchronizing movement to visual flash-like stimuli [9]. We note that performance in the HC group was lower than studies using a typical tapping synchronization interval of 600 ms to auditory tone or visual flash stimuli [12,30], most likely because of our longer tapping interval and the additional requirement to tap every two stimuli; however, those details were common across all of our synchronization conditions. The difference in performance between the unisensory visual and auditory conditions suggests that the advantage in visual synchronization that deaf individuals typically develop in response to hearing deprivation due to their heavy reliance on the visual system in daily life [12] is generally absent in the CI participants of this study. However, the trend we observed for higher unisensory visual consistency in CI users with an early deafness onset suggests that this improved visual synchronization consistency may be limited to those for whom pre-implantation deafness is both early and prolonged. Any underlying neural reorganization could also be restricted to this specific group, but a larger sample size is required to more robustly assess the validity of this trend. Additionally, most participants in this study can be considered late deafened, in contrast to the participants in the cited study [12], who were either born deaf or became deaf before the age of three years, and used American Sign Language as their primary language, which, along with early deafness, has been shown to impact visual system synchronization with visual information [31] and reaction time to visual stimuli [32].

Furthermore, our results contribute to a more nuanced understanding of the impact of cross-modal congruence and incongruence on rhythmic synchronization consistency. HC displayed improved consistency when presented with audio-visual synchronous stimuli, consistent with the idea that cross-modal timing congruence enhances sensory integration and temporal processing [33,34]. In contrast, CI users did not exhibit a significant improvement in the audio-visual synchronous condition. Additionally, CI users exhibited a significantly higher rate of unsuccessful synchronization in both the unisensory auditory and multisensory synchronous conditions, while achieving a lower rate of unsuccessful synchronization in the unisensory visual and multisensory asynchronous conditions. This finding suggests that, while auditory integration poses challenges, visual processing may still confer advantages for CI users.

Previous research demonstrated that visual stimulations elicit activation in the auditory cortex of deaf individuals [35,36]. This phenomenon appears to extend to CI users, with several studies employing fMRI and EEG suggesting an incomplete reversal of the cortical reorganization induced by deafness [37–42]. Collectively, our findings, along with those from the literature, suggest that the incomplete reversal of neural organization after implantation alters the integration of auditory sensory information with visual information. This impairment may contribute to the diminished advantage of cross-modal congruence observed in CI users. However, evidence from animal models suggests that the presence of cross-modal plasticity in higher-order auditory areas does not compromise auditory responsiveness, indicating that cross-modal reorganization may be less detrimental to neurosensory restoration than previously believed [43].

The similarity in the interference from incongruent auditory information in the asynchronous condition between CI users and HC suggests that both groups are similarly susceptible to cross-modal incongruence, which can disrupt the precision of synchronization. This result underscores that the post-implantation neural reorganization does not confer immunity to incongruent auditory information but instead affects the prioritization and integration of sensory cues.

Since the CI electrodes can only stimulate a limited number of auditory nerve fibers, the spectral resolution of the information transmitted by the CI is limited [44]. However, as already reported in the literature CI users’ temporal auditory perception remains in the same range as HC [45]. The relatively spared rhythm processing in CI users, as demonstrated in their auditory synchronization skills comparable to HC, suggests that fine-grained timing is among the aspects of auditory processing may remain intact, even following auditory deprivation and subsequent implantation. Furthermore, the observed shift in audio-visual integration among CI users, with reduced cross-modal congruence benefits, suggests that the auditory cortex may reallocate resources to prioritize auditory cues and may not efficiently integrate temporal information from visual stimuli.

Although the number of participants is typical for studies of CI users, the sample size may limit the sensitivity to determine, for example, whether auditory synchronization consistency is diminished in CI users, or whether there is an improvement in visually-paced synchronization of CI users with the addition of congruent auditory information, where both effects were non-significant in the present analyses. These open questions may be resolved in a larger study.

## Conclusion

This research aimed to determine whether CI users retain a visual synchronization advantage from their pre-implant deafness, while maintaining auditory synchronization skills comparable to those of HC, or if the neural reorganization post-implantation negates the visual synchronization advantage acquired pre-implantation. Despite impaired pitch processing [46], CI users exhibit relatively preserved rhythm processing. As expected, the auditory rhythmic synchronization abilities of CI users were found to be comparable to those of HC, corroborating existing research. Interestingly, unlike deaf individuals, CI users did not demonstrate a superior ability in synchronizing with visual rhythms, likely due to the relatively few numbers of individuals who had early prolonged pre-implantation deafness. This shift in audio-visual integration among CI users suggests that the post-deafness or post-implant reorganization of their auditory cortex might hinder the effective integration of temporal auditory input from the implant with visual information. Overall, this research provides valuable insights into the impact of cochlear implants on audio-visual synchronization abilities, suggesting a need for further investigation into the neural mechanisms involved in post-implantation reorganization and its effects on sensory integration. Despite changes in audio-visual integration following implantation, the broader perspective is that individuals with cochlear implants have access to the beautiful world of dance and rhythm, affirming their ability to engage and appreciate various forms of sensory experience.
